# Evaluation of the risk of SARS-CoV-2 infection and hospitalization in vaccinated and previously infected subjects based on real world data

**DOI:** 10.1038/s41598-023-28129-7

**Published:** 2023-02-03

**Authors:** Maicol Andrea Rossi, Tiziana Cena, Jefferson Binala, Daniela Alessi, Lorenza Scotti, Fabrizio Faggiano

**Affiliations:** 1 Department of Translational Medicine, University of Piemonte Orientale, Novara, Italy; 2 Epidemiologic Unit of the Local Health Authority of Vercelli - Osservatorio Epidemiologico, ASL Vercelli, Vercelli, Italy; 3 Department of Sustainable Development and Ecological Transition, University of Piemonte Orientale, Vercelli, Italy

**Keywords:** Viral infection, Epidemiology

## Abstract

The objective of our study was to determine the joint protective effect of a previous SARS-CoV-2 infection and vaccination on the risk of a new infection and hospitalization. Two case–control studies nested in a cohort of COVID-19 patients cared for by the Local Health Unit (LHU) of Vercelli, Italy, were performed, one to estimate the risk of infection and the second to estimate the risk of hospitalization. Each new infection and hospitalization was matched with up to 4 disease-free subjects who were the same age, sex and index date (i.e., controls). Study subjects were followed up from cohort entry date to disease outcome, end of follow-up or emigration. Vaccination was associated with a 36% (OR 0.64; 95%CI 0.62–0.66) and 90% (OR 0.10; 95%CI 0.07–0.14) reduction in the risk of infection and hospitalization, respectively. Prior infection was associated with a 65% (OR 0.35; 95%CI 0.30–0.40) and 90% (OR 0.10; 95%CI 0.07–0.14) reduction in the risk of infection and hospitalization, respectively. Vaccinated and recovered subjects showed a 63% (OR 0.37; 95%CI 0.34–0.14) and 98% (OR 0.02; 95%CI 0–0.13) reduction in the risk of infection and hospitalization, respectively. Vaccination remains an essential public health tool for preventing severe forms of COVID-19. Our study shows that vaccination or previous infection has a strong protective effect against Sars-CoV-2 hospitalization. The protective role against infection appears to be present although with a lower efficacy rate than that presented in the RCTs.

## Introduction

As of late 2019, a new disease caused by a coronavirus, named SARS-CoV-2, was identified in Wuhan, China, and soon led to the outbreak of coronavirus infectious disease 2019 (COVID-19)^1^, whose sudden spread among the general population worldwide led the World Health Organization (WHO) to declare it a pandemic on March 11th, 2020^2^.

COVID-19 patients can be affected by severe respiratory tract infections, which in some cases require hospitalization and access to intensive care units (ICUs), possibly leading to death^3,4^. Although the individual protection measures against the transmission of COVID-19 recommended by the WHO^5^ and the lockdown measures^6,7^ proved to be quite effective in reducing the transmission of the virus^8^, as of May 2022 there had been 17 million confirmed cases of COVID-19 in Italy with over 165,000 deaths^9^.

Given the enormous human, health and economic burden of the COVID-19 pandemic, researchers focused their attention on the effectiveness of natural immunity induced by SARS-CoV-2 infection in preventing reinfection. In this regard, several studies concurred that reinfection was a rare event and that patients recovered from COVID-19 had a very low risk of reinfection^10,11^. In a systematic review, the reduction in the risk of reinfection was estimated to be around 90.4 percent (*p*-value < 0.01), with an efficacy lasting up to 10 months^12^.

However, in light of the persistent incidence of severe COVID-19 cases and the high number of deaths, it soon became apparent that the much sought-after herd immunity could not simply be achieved by letting the virus spread in the population. Therefore, several effective vaccines to prevent SARS-CoV-2 infection were quickly made available.

In this scenario, the Italian COVID-19 vaccination campaign was launched on December 27th, 2020, a few days after the European Medicines Agency (EMA) approval of the mRNA-based vaccine BNT162b2 (Comirnaty). In the following months, the approval of a second mRNA vaccine, the mRNA-1273 vaccine (Spikevax), and two viral vector vaccines, the AZD1222 vaccine (Vaxzevria) and Ad26.COV2-S vaccine (Jcovden), allowed to rapidly expand the target population, achieving a high vaccination coverage. In December 2021, an adjuvanted recombinant vaccine, NVX-CoV2373 (Nuvaxovid), was also approved and included in the vaccination campaign.

The results from randomized clinical trials (RCTs) showed a decrease in symptomatic COVID-19 cases after completion of the intended vaccination cycle by more than 90% for mRNA vaccines (95% Comirnaty, 94.1% Spikevax), ~ 60–70% for viral vector vaccines (59.5% Vaxzevria; 67% Jcovden) and ~ 90% for the adjuvanted recombinant vaccine Nuvaxovid^13–18^.

The Italian vaccination plan drawn up at the end of 2020, provided for the vaccination of the entire population by summer 2021, starting with the people most at risk^19^.

Studies monitoring the incidence of COVID-19 in the general population^20,21^ as well as specific settings considered to be more at risk^22–24^ during the vaccination campaign showed a good consistency with the results from RCTs, confirming a reduction in both viral transmission and number of severe COVID-19 cases, hospitalizations and deaths.

In late 2021, the emergence of several variants of concern (VOCs) led to the hypothesis that these could reduce the effectiveness of both natural and vaccine-induced immunity. In particular, a study on the population of the Lombardy Region, Italy, showed a reduction in the effectiveness against the delta variant compared with the alpha variant (relative risk reduction of 75% *vs* 90%, respectively)^25^.

In December 2021, the SARS-CoV-2 Omicron variant (BA.1/B.1.1.529), first detected in Botswana, started to spread worldwide, leading to a rapid increase in cases throughout Europe, including Italy^26^. It soon became evident that BA.1, given the high number of spike mutations present in its genome, could evade antibody-mediated immunity arising from vaccination or previous infection with earlier variants^27^. Nevertheless, the administration of an mRNA vaccine booster dose as well as the vaccination of previously infected individuals appeared to be effective in preventing Omicron infection, although with reduced effectiveness compared with previous variants of the virus^28^.

As of May 2022, vaccination coverage in Italy for individuals over the age of 12 who had completed the primary vaccination cycle was 95.06%, while the booster dose coverage was 92.42%. With regard to subjects aged between 5 and 11 years, 62.97% had completed the primary cycle^29,30^.

In this scenario, the main objective of our study was to assess the joint protective effect of a previous SARS-CoV-2 infection and vaccination on the risk of new infections and hospitalizations for COVID-19 in the real-world setting exploiting the data available in the administrative database of the LHU of Vercelli, Piedmont, Italy. Secondary objectives were the assessment of comorbidity as a risk factor for infection or hospitalization and the evaluation of the impact of the Omicron variant on natural or vaccination-induced immunity as well as the effectiveness of the booster dose.

## Methods

### Data sources

The following administrative sources, held by the Local Health Unit (LHU) of Vercelli, Italy, were used to carry out this study: (i) population assisted by the LHU of Vercelli, containing demographic information (age and sex) and eventual exit date (death and emigration); (ii) COVID-19 platform database, containing all the information regarding the swabs performed on the population; (iii) SARS-CoV-2 vaccine database, containing information regarding the date of administration and doses of the different types of vaccines; (iv) hospital discharge records (HDRs), containing data regarding hospital admissions, including admission date and coding according to the International Classification of Diseases, 9th revision (ICD-9-CM); (v) drug registry, containing information about the provision of drugs by the regional health service with date and identification code according to the Anatomical Therapeutic Chemical (ATC) classification system; (vi) database of chronic disease exemptions from prescription charges, containing exemption codes, according to the Italian list of exempt chronic diseases, and date of release. These databases were linked through a single pseudonymised individual identification code.

### Ethical aspects

This study was conducted in the context of the epidemic surveillance function of the Epidemiologic Unit of the Local Health Authority of Vercelli. No ethical committee approval was required for the current study based on administrative databases according to the rules of the Italian Drugs Agency (http://www.agenziafarmaco.gov.it/sites/default/files/det_20marzo2008.pdf).

### Study design and setting

We performed two case–control studies nested in the cohort of citizens assisted by the LHU of Vercelli. As of December 27th, 2020, the starting date of the SARS-CoV-2 vaccination campaign in Italy, all study subjects were alive.

In the first study, cases were all the 31,832 nasopharyngeal swab positive for SARS-CoV-2, while in the second study, cases were all the 911 subjects hospitalized for SARS-CoV-2 in the population assisted by the LHU of Vercelli.

Each case was matched with up to 4 controls of the same age and sex who were disease free at the date of the outcome under study (index date) of the corresponding case. In the first study the controls were 127,238, whereas in the second study they were 3646.

Subjects were followed up from the cohort entry date to the occurrence of any of the following events: outcome (i.e., infection or hospitalization, depending on the study), death, emigration or end of follow-up (May 8th, 2022 for the first study; March 1st, 2022 for the second study).

### Case definition

For the study on infection, cases were defined by the first nasopharyngeal swab for SARS-CoV-2, either molecular or antigenic, with a positive result after December 27th, 2020. A positive swab found less than 90 days after a previous positive swab diagnosed before December 27th, 2020 were excluded from the study.

For the study on hospitalization, cases were defined by the first hospitalization for COVID-19 occurring after December 27th, 2020. The hospitalizations were identified through HDR codes related to COVID-19 disease (043.11, 043.21, 043.31, 480.41, 518.91, 519.71). Hospitalization was stratified on the basis of severity into two categories: ‘severe’ and ‘non-severe’. The ‘severe’ subjects were identified by HDR codes indicative of a particular severity of illness like sepsis, septic shock, respiratory failure,… (518.81, 995.91, 995.92, 286.6, 348.31, 570, from 584.5 to 584.9, from 428.0 to 428.9, 785.52).

### Exposure assessment

Exposure was defined as either previous SARS-Cov-2 infection or vaccination against SARS-Cov-2.

Previous infection was defined as a SARS-CoV-2 positive swab recorded between March 1st, 2020 and December 26th, 2020.

The vaccination status of cases and controls was defined in accordance with the Italian guidelines, which state that a subject is to be considered fully vaccinated 14 days after the administration of the second dose of vaccine or a single dose if the subject has previously been positive or has been vaccinated with the Jcovden vaccine (Johnson & Johnson). Subjects who received a two-dose primary cycle following Sars-CoV-2 infection were considered together with subjects who received a primary cycle with a single dose after infection. In Italy, a booster dose can be administered from 120 days after completion of the primary cycle.

The exposure status was analyzed as follows: (i) previous SARS-Cov-2 infection and vaccination as two independent dichotomous variables; (ii) previous SARS-Cov-2 infection and vaccination jointly as a four level categorical variable, defined as no previous infection and no vaccination, previous infection only, vaccination only, previous infection and vaccination; and (iii) vaccination status considering four categories: no vaccination, cycle completed by less than 120 days, cycle completed by more than 120 days and booster dose.

Reinfection is defined by the Italian Ministry of Health as a second SARS-CoV-2 positive swab at least 90 days after the first positivity or less than 90 days if caused by two different variants of the virus identified through laboratory methods. Not having sequencing data available for our study, we only adopted the former criterion.

To study the effect of the Omicron variant on the effectiveness of the infection and of the vaccination, the analysis were performed stratifying by time periods, according to the calendar of infections: from the beginning of the epidemic, until December 2021, and from December to the end of the observation period.

### Definition of the vulnerability index

An index combining chronic conditions and their impact on the subjects’ health was created to assess the vulnerability status of our study subjects.

The presence of chronic disease among cases and controls was assessed in 2019, before the onset of the SARS-CoV-2 pandemic, using administrative databases, exemptions for prescription charges, hospital discharge and drug prescriptions.

The list of chronic conditions considered by Corrao et al.^25^ was retrieved. For each disease included in this list, the exemption, ATC and ICD-9-CM codes required to classify the different diseases were identified. A subject was defined as affected by one of the selected chronic diseases if he/she had at least one among the following criteria: (i) a prescription charge exemption, (ii) a hospitalization recorded in either the principal diagnosis or secondary diagnoses, or (iii), at least 3 prescriptions for specific drugs. The prescription charge exemption codes, ICD-9-CM and ATC used are reported in the Supplementary (Table S2).

In order to take into account the impact of chronic conditions on the risk of severe forms of COVID-19, a vulnerability index was elaborated based on the Italian Ministry of Health chronic diseases classification contained in the COVID-19 vaccination plan^19^. To each condition was assigned a score from 1 (not severe condition) to 3 (severe condition). All subjects were classified according to the most severe disease they are affected as: “3” = “extremely vulnerable” individuals; “2” = “frail” subjects; “1” = having chronic diseases that do not lead to increased risk of severe COVID-19, “0” = not having chronic diseases. Subjects with vulnerability index “0” and “1” were classified as “non-frail subjects”.

### Statistical analysis

The subject’s characteristics and exposures were described using absolute and relative frequencies for categorical variables and mean and standard deviation (SD) or median and interquartile range (IQR) for numerical ones, as appropriate, according to the outcome status.

Univariable conditional logistic-regression models were applied to estimate the odds ratio (OR) and 95% confidence interval (95%CI) of the association between exposures, vulnerability index and SARS-CoV-2 infection or hospitalization. Moreover, multivariable conditional logistic-regression models were applied to estimate the association between the interaction between vaccination and prior infection and the risk of SARS-CoV-2 infection or hospitalization adjusted for the vulnerability index. The latter model was applied to the overall set of cases and controls and separately for those occurring before December 1st, 2021 and after to assess the exposures effect on different variants of COVID-19, assuming that cases occurring after December 1st, 2021 were due to the Omicron variant. Finally, considering only the outcomes of the cases occurring after December 1st, 2021, multivariable conditional logistic regression models were fitted for exposure, time since second dose administration and booster.

All the analyses were conducted using SAS 9.4 (Cary, NC, USA). For hypothesis testing, *p* < 0.05 (two-tailed) was considered statistically significant.

## Results

### Descriptive features of the study population

During the study period (from December 27th, 2020 to May 8th, 2022), the SARS-CoV-2 pandemic led to 31,832 subjects testing positive for SARS-CoV-2 among the population cared for by the LHU of Vercelli. Of these, 46.8% were male, the mean age was 41.4 (SD: 22.5) years, 57% were fully vaccinated, and 2.2% had already had a positive swab before December 27th, 2020. The mean follow-up time was 341.7 (SD: 130.8) days. Among fully vaccinated, 75.3% received Comirnaty vaccine (73.9% among controls), 13.2% Spikevax vaccine (14.4% among controls), 9.6% Vaxzevria vaccine (9.5% among controls), 1.9% Jcovden vaccine (2.2% among controls), and 0.01% Nuvaxovid vaccine (0.01% among controls); 40.8% received the booster dose (47.9% among controls). During the same period, 911 subjects, representing 2.9% of all positive cases, were hospitalized for COVID-19-related issues. They were predominantly males (56.2%), with a mean age of 69.3 (SD: 14.9) years. Only 13.3% of them had completed the primary vaccine course, and only 0.4% had been previously found positive. The mean follow-up time was 154.4 (SD: 132.6) days (Table 1). Among fully vaccinated, 73.8% received Comirnaty vaccine (73.4% among controls), 4.1% Spikevax vaccine (9.5% among controls), 16.4% Vaxzevria vaccine (15.6% among controls), 5.7% Jcovden vaccine (1.5% among controls). Among cases and controls no one received the Nuvaxovid vaccine; 34.4% received the booster dose (46.9% among controls). Considering the severity of hospitalization, among the 911 hospitalized cases, 659 (or 72.3%) were classified as ‘severe’.

**Table 1:** Distribution of cases and controls’ characteristics of in the two studies, odds ratios (OR) and corresponding 95% confidence intervals (95%CI) derived from univariable conditional logistic regression.

Characteristics of the population	Infection			Hospitalization		
	Controls N = 127,328	Cases N = 31,832	OR (95%CI)	Controls N = 3646	Cases N = 911	OR (95%CI)
	N (%)	N (%)		N (%)	N (%)	
Male	59,620 (46.80)	14,905 (46.80)	–	2050 (56.20)	512 (56.20)	–
Age, mean (SD)	41.40 (22.50)	41.40 (22.50)	–	69.30 (14.90)	69.30 (14.90)	–
Vaccinated	79,319 (62.30)	18,149 (57.00)	0.65 (0.63–0.67)	1008 (27.60)	122 (13.40)	0.11 (0.08–0.16)
Previously infected	5570 (4.40)	706 (2.20)	0.49 (0.46–0.53)	164 (4.50)	5 (0.50)	0.12 (0.05–0.28)
Immunization status						
Unvaccinated and not previously infected	46,200 (36.30)	13,492 (42.40)	1 (ref)	2525 (69.30)	785 (86.20)	1 (ref)
Vaccinated and not previously infected	75,558 (59.30)	17,634 (55.40)	0.64 (0.62–0.66)	957 (26.20)	121 (13.30)	0.11 (0.08–0.16)
Unvaccinated and previously infected	1809 (1.40)	191 (0.60)	0.35 (0.30–0.41)	113 (3.10)	4 (0.40)	0.10 (0.04–0.28)
Vaccinated and previously infected	3761 (3.00)	515 (1.60)	0.38 (0.34–0.41)	51 (1.40)	1 (0.10)	0.02 (0.00–0.15)
Subjects with chronic disease in 2019	39,597 (31.10)	10,211 (32.10)	1.06 (1.03–1.10)	2332 (64.00)	670 (73.50)	1.71 (1.43–2.04)
Vulnerability index						
Non-frail subjects (severity index 0 or 1)	90,049 (70.70)	22,231 (69.80)	1 (ref)	1385 (38.00)	263 (28.90)	1 (ref)
Frail subjects (severity index 2)	24,747 (19.40)	6304 (19.80)	1.05 (1.01–1.08)	1407 (38.60)	375 (41.10)	1.51 (1.26–1.82)
Extremely vulnerable subjects (severity index 3)	12,532 (9.80)	3297 (10.40)	1.09 (1.04–1.14)	854 (23.40)	273 (30.00)	1.83 (1.50–2.25)

As for the subjects who needed hospitalization, 73.5% had at least one chronic pathology. Of these, 3% (n = 22) had a vulnerability index of 1, 56% were classified as frail, and 41% were deemed extremely vulnerable.

The prevalence of each chronic disease among cases and controls for both case–control studies is reported in Table S1 (see Supplementary Material) as well as the OR and 95%CI for the association between chronic disease and SARS-CoV-2 infection and hospitalization.

### Effect of natural and induced immunity on SARS-CoV-2 reinfection

Compared with controls, exclusively vaccinated subjects were 36% less likely to be infected than unvaccinated subjects (OR 0.64; 95%CI 0.62–0.66). For hospitalizations this probability was further decreased by 89% (OR 0.11; 95%CI 0.08–0.16). On the other hand, subjects with prior SARS-CoV-2 infection displayed a 65% reduced risk of acquiring a second infection and a 90% reduced risk of experiencing hospitalization. Similarly, previously positive subjects who had undergone vaccination had a 62% and 98% reduced risk of infection and hospitalization, respectively.

Compared to individuals with lower vulnerability indexes, subjects classified as “frails” and “extremely vulnerable” showed increased risk of hospitalization by 51% and 83%, respectively. Frailty had a lower, albeit statistically significant, impact on infection with an increased risk of 5% and 9%, respectively (Table 1).

**Figure 1:**
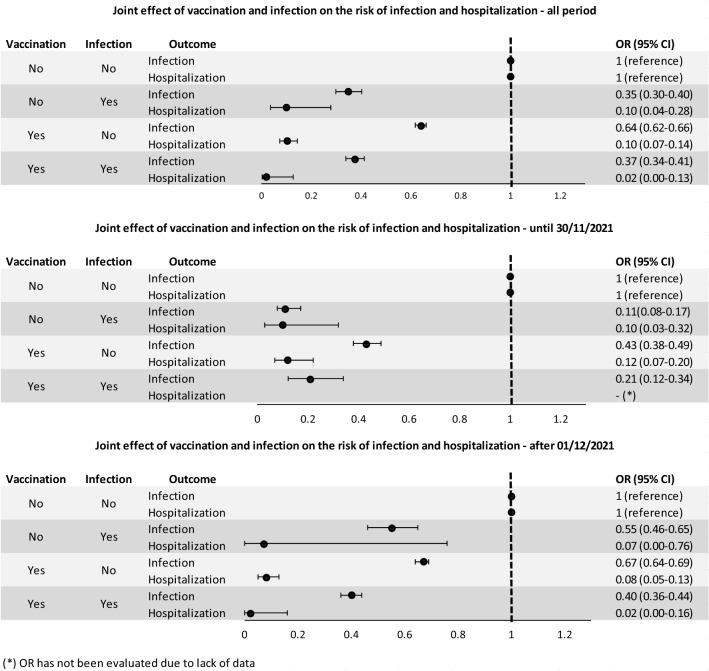
Multivariable conditional logistic-regression models. Odds ratios (OR) and 95% confidence intervals (95%CI) for the association between joint effect of vaccination and previous infection and risk of new infection and hospitalization. Results are stratified by wave of infection and adjusted by vulnerability index (Datas on Table S3).

Evaluating the joint effect of vaccination and previous infection by multivariable analysis adjusted for vulnerability index, we found that the risk of infection and hospitalization were respectively reduced by 65% and 90% in previously infected unvaccinated individuals, 36% and 90% in vaccinated individuals who had not been previously infected, and 63% and 98% in previously infected and vaccinated individuals (Fig. 1).

To evaluate the effect of the Omicron variant, which become prevalent in Italy in December 2021, we divided the study period in two parts: before and after December 1st, 2021. Multivariable analysis of both periods showed that the reduction in the risk of infection in previously infected subjects went from 89% (first period, before Omicron) to 45% (second period, with Omicron). Similarly, the effect of vaccination on reducing the risk of infection went from 57% (before Omicron) to 33% (with Omicron). In vaccinated and COVID-19 recovered subjects, the risk of infection decreased from 79% (before Omicron) to 60% (with Omicron). Regarding the risk of hospitalization, the results had very wide confidence intervals due to the small sample size and were essentially similar for the two study periods (Fig. 1). OR and corresponding 95%CI reported in Fig. 1 as well as those of the vulnerability index are reported in Table S3 (see Supplementary Material).

In the period after December 1st, 2021, we also evaluated the effect of vaccination, adjusted for the presence of chronicity and any previous infection and stratified by vaccination status. Among the infection cases included in the study during this period (25,146 subjects with 100,584 controls), 30% were unvaccinated, 13% and 27% had completed the primary course for less or more than 120 days, respectively, with a respective mean time of 79.1 (SD: 31.4) and 162.6 (SD: 30.7) days, and 30% had already received the booster dose for a mean time of 61.2 (SD: 44.6) days.

Concerning the hospitalization cases (190 subjects with 760 controls), 53% of them were unvaccinated, 4% and 22% had completed the primary cycle by less than or more than 120 days, respectively, with a respective mean time of 76.2 (SD: 32) and 175.7 (SD: 39.4), and 21% had received the booster dose by an average of 45.3 (SD: 27) days.

Multivariable conditional logistic-regression analysis showed that administration of the booster dose resulted in a greater reduction in the risk of infection (52%) than that observed in subjects undergoing the primary cycle alone—27% and 11% within or after 120 days after completion of the primary vaccine cycle, respectively (Table 2). Likewise, the booster dose resulted in a reduction in the risk of hospitalization among subjects with the primary vaccine cycle completed by more or less than 120 days (96% *vs* 80% and 82%, respectively).

**Table 2:** Odds ratios (OR) and 95% confidence intervals (95%CI) for the association between joint effect of vaccination and previous infection and risk of new infection and hospitalization occurred after December 1st, 2021.

Vaccination status	Infection	Hospitalization
	OR (95% CI)*	OR (95% CI)*
No vaccination	1 (ref)	1 (ref)
Cycle completed by > 120 days	0.89 (0.86–0.93)	0.18 (0.10–0.31)
Cycle completed by ≤ 120 days	0.73 (0.70–0.77)	0.20 (0.08–0.51)
Booster dose	0.48 (0.46–0.50)	0.04 (0.02–0.07)

*Adjusted for age, sex (matching) and vulnerability index.

With regard to the severity of hospitalization, it is interesting to point out that in the period before December 1st, 2021, of the 6686 cases of infection, 721 subjects (corresponding to 10.8%) required hospitalization and among these 521 (corresponding to 72.3%) were classified as “serious”.

In the period after December 1st, 2021, of the 25,146 cases of infection, 190 subjects (corresponding to 0.7%) required hospitalization, of whom 138 (corresponding to 72.6%).

## Discussion

The objective of our study was to assess the joint effect of vaccination and natural immunity conferred by infection on the risk of new infection and hospitalization among the general population, taking also into account the presence of chronic diseases, exploiting data of the administrative database of the LHU of Vercelli.

The results of our study, based on real world data, show that the protection conferred by vaccination from infection in our study population appears to be much lower than that observed in RCTs^13–18^. This was partly expected because we assessed different waves of epidemics resulting in variable levels of vaccine responsiveness and enrolled subjects with different times since vaccination^25–27^. On the other hand, in spite of the aforementioned factors, we found that protection from hospitalization is similar to that reported by RCTs^13–18^.

Focusing on the last observation period—from December 2021 on—characterized by the emergence of the Omicron variant, which led to a reduction in the effectiveness of vaccines in preventing infection and the need to administer a booster dose to the population, our results show a sharp decrease in protection from infection (OR 0.67; 95%CI 0.64–0.69) compared to that observed in RCTs (vaccine efficacy 95%; 95%CI 90.3–97.6), whereas the protection from hospitalization appears to be more persistent (OR 0.08; 95%CI 0.05–0.13)^15,28^.

Another original result of our research is the effect of natural immunization on reinfection. Before the emergence of the Omicron variant, such acquired immunity provided a very long-lasting protection, persisting for more than 1 year^31,32^. However, during the Omicron wave, the protection from reinfection seems to decrease among recovered individuals, whereas the protection from hospitalization persists for a longer time. Interestingly, the risk of hospitalization after recovery from infection does not vary significantly throughout the study period. However, these results are influenced by the small sample sizes, especially in the category of previously positive unvaccinated and previously positive vaccinated. Despite the low statistical power of our analysis in these groups, from a clinical standpoint it suggests that a previous infection combined with vaccination confers a substantial protective effect, confirming that vaccination remains a key tool with which to tackle the COVID-19 pandemic. Indeed, the hospitalization rates during the last pandemic wave (December 2021-January 2022) always remained below alarm levels (0.7% versus 10.8% in the previous period) despite a very high number of daily cases, allowing normal life to continue without the need to go to extreme measures, such as lockdowns. With regard to the severity of the clinical conditions of those hospitalized, although the percentage of those infected who required hospitalization decreased, among them the percentage of ‘severe’ hospitalizations did not. This result may be explained by a selection bias whereby the subjects hospitalized are always those with the most severe pathology. Looking at the absolute numbers, it is clear that the Omicron variant has had less impact on hospital facilities, with far fewer serious cases than the previous variants.

One of the strengths of our study is undoubtedly the large number of cases and, consequently controls analyzed. Having access to databases related to the presence of chronic diseases allowed us to adjust our estimates for all those comorbidities associated with increased risk of severe COVID-19.

Some possible biases may affect the results of our study. Specifically, we know that in 2020, especially during the early months of the pandemic, access to nasopharyngeal swab was limited, and that for this reason many individuals became infected with SARS-CoV-2 without a laboratory diagnosis. This may have led us to underestimate the protective effect of prior infection, because some controls could have been infected. The protective effect of prior infection may have been overestimated because it was calculated only on individuals who survived the first infection. Indeed, these subjects were likely to have a more efficient immune system than that of more vulnerable ones who were at greater risk of infection and death. Another limitation of our study is the absence of data on infection-related symptoms. We decided not to include these data in our analysis due to their poor and variable quality in the available administrative sources. Furthermore, we chose not to distinguish the effects induced by different types of vaccine because, by doing so, we would have greatly reduced the size of our sample. However, from previous studies we knew that these vaccines were equally effective in preventing severe COVID-19 but not infection^13–18^. We should also consider that more than 90% of the population under study received mRNA-based vaccines^33^ and that only these types of vaccine were used for the booster dose. Lastly, we were unable to assess the risk of death from COVID-19 because the Italian mortality statistics are released by the Italian Institute of Statistics (ISTAT) with a delay of almost 3 years.

In conclusion, our findings confirm that vaccination is still the best public health tool with which to keep in check the SARS-CoV-2 pandemic, especially its related hospitalizations.

Previous infections appear to have a similar effect to that of vaccination, conferring a better protection against hospitalization than against reinfection. However, given the fading effect of current vaccines against infection, it is of the utmost importance to continue monitoring the evolution of the pandemic in the upcoming months to understand whether the appearance of new variants or increased time since vaccination leads to a further reduction in vaccine-induced protection, which would raise the need for an additional booster dose.

## Supplementary Information

Supplementary Tables.

## Data Availability

The datasets generated and/or analysed during the current study are not publicly available because the administrative databases used for the analysis are owned by the Piedmont Region, the data controller to whom any requests for data sharing should be addressed.
