# Supplementary material for: Evaluation of the risk of SARS-CoV-2 infection and hospitalization in vaccinated and previously infected subjects based on real world data
Source: Sci Rep. 2023 Feb 3;13:2018. doi: 10.1038/s41598-023-28129-7 (PMC9897610; doi:10.1038/s41598-023-28129-7)
Supplement: Supplementary file 1 — Supplementary Tables. [file 41598_2023_28129_MOESM1_ESM.docx]

**Supplementary material**

**Evaluation of the risk of SARS-CoV-2 Infection and Hospitalization in Vaccinated and Previously Infected Subjects Based on Real World Data.**

[Table S1 – Prevalence and Risk of Infection and Hospitalization by Chronic Diseases among Cases and Controls. Univariate Conditional Logistic-Regression Models 2](#_Toc110425065)

[Table S2 – Ticket exemptions, ICD-9-CM and ATC Codes used to identify subjects with chronic diseases 4](#_Toc110425066)

[Table S3 - Effect of Vaccination and Infection on the Risk of New Infection and Hospitalization. Multivariate Conditional Logistic-Regression Analysis, Adjusted by Vulnerability Index 9](#_Toc110425067)

**Table S1 – Prevalence and Risk of Infection and Hospitalization by Chronic Diseases among Cases and Controls. Univariate Conditional Logistic-Regression Models**

| **Chronic diseases** | **Infection** | | | **Hospitalization** | | |
| --- | --- | --- | --- | --- | --- | --- |
|  | **Controls**  **N=127328** | **Cases**  **N=31832** | **OR (95% IC)*** | **Controls**  **N=3646** | **Cases**  **N=911** | **OR (95% IC)*** |
|  | **N (%)** | **N (%)** |  | **N (%)** | **N (%)** |  |
| *HIV infection* | 229 (0.2) | 37 (0.1) | 0.65 (0.46-0.91) | 0 (0.0) | 2 (0.2) | NE |
| *Tuberculosis and other infectious and parasitic diseases* | 53 (0.0) | 15 (0.0) | 1.13 (0.64-2.01) | 3 (0.1) | 1 (0.1) | 1.33 (0.14-12.82) |
| *Solid malignancies and neoplasms of lymphatic and hematopoietic tissue* | 5031 (4.0) | 1313 (4.1) | 1.05 (0.98-1.12) | 350 (9.6) | 100 (11) | 1.16 (0.92-1.47) |
| *Benign neoplasm and carcinoma in situ* | 404 (0.3) | 112 (0.4) | 1.11 (0.90-1.37) | 10 (0.3) | 5 (0.5) | 2.00 (0.68-5.85) |
| *Hypothyroidism* | 5233 (4.1) | 1418 (4.5) | 1.09 (1.03-1.16) | 273 (7.5) | 71 (7.8) | 1.05 (0.79-1.38) |
| *Hyper and hypoparathyroidism* | 47 (0.0) | 12 (0.0) | 1.02 (0.54-1.93) | 0 (0.0) | 0 (0.0) | NE |
| *Diabetes without insulin therapy* | 5412 (4.3) | 1301 (4.1) | 0.96 (0.90-1.02) | 446 (12.2) | 141 (15.5) | 1.33 (1.08-1.64) |
| *Insulin therapy* | 1041 (0.8) | 285 (0.9) | 1.10 (0.96-1.25) | 78 (2.1) | 36 (4.0) | 1.89 (1.26-2.84) |
| *Dyslipidemia* | 8981 (7.1) | 2281 (7.2) | 1.02 (0.97-1.08) | 823 (22.6) | 232 (25.5) | 1.19 (1.00-1.42) |
| *Obesity* | 91 (0.1) | 18 (0.1) | 0.79 (0.48-1.31) | 5 (0.1) | 1 (0.1) | 0.80 (0.09-6.85) |
| *Weight loss* | 16 (0.0) | 3 (0.0) | 0.75 (0.22-2.57) | 1 (0.0) | 0 (0.0) | NE |
| *Disorders of fluid, electrolyte, and acid-base balance* | 129 (0.1) | 36 (0.1) | 1.12 (0.77-1.62) | 11 (0.3) | 5 (0.5) | 1.82 (0.63-5,23) |
| *Gout* | 1192 (0.9) | 355 (1.1) | 1.20 (1.06-1.36) | 142 (3.9) | 54 (5.9) | 1.56 (1.13-2.17) |
| *Other disorders of endocrine, nutritional and metabolic systems* | 2623 (2.1) | 718 (2.3) | 1.10 (1.01-1.2) | 83 (2.3) | 18 (2) | 0.86 (0.51-1.45) |
| *Disorders involving the immune system* | 2 (0.0) | 1 (0.0) | 2.00 (0.18-22.06) | 2 (0.1) | 0 (0.0) | NE |
| *Coagulation defects* | 22 (0.0) | 4 (0.0) | 0.73 (0.25-2.11) | 0 (0.0) | 0 (0.0) | NE |
| *Autoimmune hemolytic anemias, other anemias, anemias* | 1467 (1.2) | 402 (1.3) | 1.1 (0.98-1.23) | 112 (3.1) | 42 (4.6) | 1.54 (1.07-2.23) |
| *Other diseases of the blood and blood-forming organs* | 49 (0.0) | 17 (0.1) | 1.39 (0.80-2.41) | 0 (0.0) | 0 (0.0) | NE |
| *Dementia/Alzheimer* | 249 (0.2) | 77 (0.2) | 1.24 (0.96-1.61) | 27 (0.7) | 10 (1.1) | 1.48 (0.72-3.06) |
| *Psychosis* | 1028 (0.8) | 213 (0.7) | 0.83 (0.71-0.96) | 52 (1.4) | 22 (2.4) | 1.71 (1.03-2.84) |
| *Depression* | 5207 (4.1) | 1451 (4.6) | 1.13 (1.06-1.20) | 309 (8.5) | 106 (11.6) | 1.43 (1.13-1.81) |
| *Bipolar disorders* | 206 (0.2) | 36 (0.1) | 0.70 (0.49-1.00) | 6 (0.2) | 2 (0.2) | 1.33 (0.27-6.61) |
| *Alcohol abuse* | 130 (0.1) | 24 (0.1) | 0.74 (0.48-1.14) | 5 (0.1) | 1 (0.1) | 0.80 (0.09-6.85) |
| *Drug addiction* | 173 (0.1) | 19 (0.1) | 0.44 (0.27-0.70) | 3 (0.1) | 0 (0.0) | NE |
| *Anxiety* | 32 (0.0) | 3 (0.0) | 0.38 (0.11-1.22) | 0 (0.0) | 0 (0.0) | NE |
| *Other mental disorders* | 201 (0.2) | 35 (0.1) | 0.70 (0.49-1.00) | 5 (0.1) | 1 (0.1) | 0.80 (0.09-6.85) |
| *Parkinson’s disease and parkinsonism* | 507 (0.4) | 148 (0.5) | 1.17 (0.97-1.41) | 54 (1.5) | 22 (2.4) | 1.67 (1.00-2.77) |
| *Multiple sclerosis* | 267 (0.2) | 58 (0.2) | 0.87 (0.65-1.15) | 4 (0.1) | 2 (0.2) | 2.00 (0.37-10.92) |
| *Epilepsy and recurrent seizures* | 1657 (1.3) | 393 (1.2) | 0.95 (0.85-1.06) | 75 (2.1) | 29 (3.2) | 1.56 (1.01-2.41) |
| *Glaucoma* | 1817 (1.4) | 496 (1.6) | 1.10 (0.99-1.21) | 166 (4.6) | 53 (5.8) | 1.31 (0.94-1.81) |
| *Disorders of the eye and adnexa* | 0 (0.0) | 0 (0.0) | NE | 0 (0.0) | 0 (0.0) | NE |
| *Arrhythmia* | 2049 (1.6) | 593 (1.9) | 1.17 (1.06-1.29) | 199 (5.5) | 67 (7.4) | 1.40 (1.04-1.87) |
| *Valvular diseases* | 431 (0.3) | 128 (0.4) | 1.19 (0.98-1.45) | 41 (1.1) | 12 (1.3) | 1.17 (0.61-2.24) |
| *Vascular diseases* | 478 (0.4) | 133 (0.4) | 1.12 (0.92-1.35) | 52 (1.4) | 11 (1.2) | 0.84 (0.44-1.63) |
| *Cerebrovascular diseases* | 550 (0.4) | 154 (0.5) | 1.12 (0.94-1.34) | 52 (1.4) | 20 (2.2) | 1.54 (0.92-2.58) |
| *Hypertension* | 11620 (9.1) | 3003 (9.4) | 1.05 (1.00-1.10) | 1038 (28.5) | 274 (30.1) | 1.09 (0.92-1.28) |
| *Coronary and peripheral vascular disease* | 1093 (0.9) | 310 (1.0) | 1.14 (1.00-1.29) | 90 (2.5) | 35 (3.8) | 1.60 (1.07-2.39) |
| *Oral anticoagulant agents* | 1705 (1.3) | 484 (1.5) | 1.15 (1.03-1.28) | 191 (5.2) | 73 (8) | 1.62 (1.21-2.17) |
| *Other diseases of the circulatory system* | 1829 (1.4) | 501 (1.6) | 1.10 (1.00-1.22) | 156 (4.3) | 55 (6) | 1.45 (1.05-2.01) |
| *Chronic obstructive pulmonary disease, asthma, chronic respiratory disease* | 2974 (2.3) | 907 (2.8) | 1.23 (1.14-1.32) | 88 (2.4) | 53 (5.8) | 2.48 (1.75-3.5) |

*****reference: subject without the chronic condition, NE: not estimable due to lack of exposed subject

**Table S1 continued**

| **Chronic diseases** | **Infection** | | | **Hospitalization** | | |
| --- | --- | --- | --- | --- | --- | --- |
|  | **Controls**  **N=127328** | **Cases**  **N=31832** | **OR (95% IC)*** | **Controls**  **N=3646** | **Cases**  **N=911** | **OR (95% IC)*** |
|  | **N (%)** | **N (%)** |  | **N (%)** | **N (%)** |  |
| Acute respiratory infections | 0 (0.0) | 0 (0.0) | NE | 0 (0.0) | 0 (0.0) | NE |
| Cystic fibrosis | 117 (0.1) | 32 (0.1) | 1.09 (0.74-1.62) | 6 (0.2) | 5 (0.5) | 3.65 (1.04-12.75) |
| Other diseases of the respiratory system | 831 (0.7) | 248 (0.8) | 1.20 (1.04-1.38) | 59 (1.6) | 29 (3.2) | 2.00 (1.28-3.14) |
| Liver cirrhosis and other liver chronic diseases | 1012 (0.8) | 261 (0.8) | 1.03 (0.90-1.18) | 47 (1.3) | 12 (1.3) | 1.02 (0.54-1.93) |
| Inflammatory bowel diseases (Ulcerative colitis and Chron’s disease) | 697 (0.5) | 187 (0.6) | 1.07 (0.91-1.26) | 37 (1) | 11 (1.2) | 1.19 (0.61-2.33) |
| Chronic and acute pancreatitis | 75 (0.1) | 20 (0.1) | 1.07 (0.65-1.75) | 5 (0.1) | 1 (0.1) | 0.80 (0.09-6.85) |
| Other diseases of the digestive system | 620 (0.5) | 152 (0.5) | 0.98 (0.82-1.17) | 15 (0.4) | 3 (0.3) | 0.80 (0.23-2.76) |
| Chronic kidney disease | 454 (0.4) | 155 (0.5) | 1.37 (1.14-1.65) | 41 (1.1) | 15 (1.6) | 1.48 (0.81-2.70) |
| Other kidney disorders | 114 (0.1) | 39 (0.1) | 1.37 (0.95-1.98) | 5 (0.1) | 5 (0.5) | 4.00 (1.16-13.82) |
| Other diseases of the genitourinary system | 757 (0.6) | 186 (0.6) | 0.98 (0.84-1.15) | 43 (1.2) | 17 (1.9) | 1.60 (0.90-2.82) |
| Diseases of the skin and subcutaneous tissues, including not rheumatoid psoriasis | 414 (0.3) | 105 (0.3) | 1.01 (0.82-1.26) | 24 (0.7) | 11 (1.2) | 1.90 (0.91-3.96) |
| Autoimmune disease (Rheumatoid arthritis, rheumatoid psoriasis, anchylosing spondylitis, systemic sclerosis, systemic lupus erythematosus) | 506 (0.4) | 155 (0.5) | 1.23 (1.02-1.47) | 15 (0.4) | 11 (1.2) | 2.93 (1.35-6.39) |
| Other diseases of the musculoskeletal system and connective tissue | 103 (0.1) | 31 (0.1) | 1.20 (0.81-1.80) | 3 (0.1) | 3 (0.3) | 4.00 (0.81-19.82) |
| Symptoms, signs and ill-defined conditions | 0 (0.0) | 0 (0.0) | NE | 0 (0.0) | 0 (0.0) | NE |
| Transplantation | 304 (0.2) | 86 (0.3) | 1.13 (0.89-1.44) | 17 (0.5) | 5 (0.5) | 1.18 (0.43-3.19) |
| Chronic pain | 789 (0.6) | 223 (0.7) | 1.13 (0.98-1.32) | 72 (2) | 24 (2.6) | 1.35 (0.84-2.17) |
| Corticosteroids | 2098 (1.6) | 569 (1.8) | 1.09 (0.99-1.19) | 116 (3.2) | 51 (5.6) | 1.80 (1.29-2.53) |
| Hormone therapy (oral contraceptives or replacement hormone therapy) | 109 (0.1) | 22 (0.1) | 0.81 (0.51-1.28) | 0 (0.0) | 1 (0.1) | NE |
| Rare Disease | 1087 (0.9) | 299 (0.9) | 1.01 (0.97-1.25) | 25 (0.7) | 8 (0.9) | 1.28 (0.58-2.84) |

*****reference: subject without the chronic condition, NE: not estimable due to lack of exposed subject

**Table S2 – Ticket exemptions, ICD-9-CM and ATC Codes used to identify subjects with chronic diseases**

| **Diagnostic Categories** | **Disease** | **Vulnerability index** | **Ticket exemption code** | **ICD-9-CM** | **ATC** |
| --- | --- | --- | --- | --- | --- |
| **Infectious and parasitic diseases** | *HIV infection* | **2** | 020.042, 020.V08 | 042, V08 | J05AB14, J05AE, J05AF01, J05AF02, J05AF04, J05AF05, J05AF06, J05AF09, J05AG, J05AR, J05AX07, J05AX08, J05AX09, J05AX12 |
|  | *Tuberculosis and Other infectious and parasitic diseases* | **2** | 055.010, 055.011, 055.012, 055.013, 055.014, 055.015, 055.016, 055.017, 055.018 | 010-018, 045, 090-104, 135-139 | J04AB |
| **Neoplasms** | *Solid malignancies and Neoplasm of lymphatic and hematopoietic tissue* | **3** | 048 | 140-165, 170-176, 179-199, 200-208, V58.0, V58.1, 192.2 | L01, L03AC, L02BA01, L02BA02, L02BG02, L02BG03, L02BG04, L02BG06, L02BB01, L02BB03, L02AE02, L02AE04, L02AB01 |
|  | *Benign neoplasm and carcinoma in situ* | **1** |  | 210-234 |  |
| **Endocrine, nutritional and metabolic diseases, and immunity disorders** | *Hypothyroidism* | **2** | 027.243, 027.244 | 243, 244 | H03A, H03B |
|  | *Hyper and hypoparathyroidism* | **2** | 026.252.0, 026.252.1 | 252.0, 252.1 |  |
|  | *Diabetes without insulin therapy* | **2** | 013.250 | 250, 648.0, 357.2, 362.0, 366.41 | A10B |
|  | *Insulin therapy* | **2** |  |  | A10A |
|  | *Dyslipidemia* | **2** | 025.272.0, 025.272.2, 025.272.4 | 272.0, 272.2, 272.4 | C10 |
|  | *Obesity* | **3** |  | 278.0 |  |
|  | *Weight loss* | **2** |  | 260-263 |  |
|  | *Disorders of fluid, electrolyte, and acid-base balance* | **1** |  | 276 |  |
|  | *Gout* | **1** |  | 274 | M04AC01, M04AA, M04AB |
|  | *Other disorders of endocrine, nutritional and metabolic diseases* | **1** | 001.253.0, 012.253.5, 022.255.4, 032.255.0, 035.242.0, 035.242.1, 035.242.2, 035.242.3, 039.253.3, 056.245.2 | 240-242, 245, 246, 249, 251, 252.8, 252.9, 253-259, 270, 271, 272.0, 272.1, 272.3, 272.5-272.9, 273, 275, 277.1-277.9, 278.1-278.8 |  |
|  | *Disorders involving the immune mechanisms* | **3** |  | 279 |  |

**Table S2 continued**

| **Diagnostic Categories** | **Disease** | **Vulnerability index** | **Ticket exemption code** | **ICD-9-CM** | **ATC** |
| --- | --- | --- | --- | --- | --- |
| **Diseases of the blood and blood-forming organs** | *Coagulation defects* | **2** |  | 286 | B02B |
|  | *Autoimmune hemolytic anemias, Other anemias, Anemias only tracked from drug therapy* | **2** | 003.283.0 | 280-285 | B03A, B03B, B03XA01, L03AA |
|  | *Other diseases of the blood and blood-forming organs* | **2** |  | 287-289 |  |
| **Mental disorders** | *Dementia / Alzheimer* | **1** | 011.290.0, 011.290.1, 011.290.2, 011.290.4, 029.331.0 | 290.0-290.4, 331.0 | N06DA, N06DX01 |
|  | *Psychosis* | **2** | 044.295.0, 044.295.1, 044.295.2, 044.295.3 | 295, 297, 298.2-298.9, 299.1 | N05AD, N05AA, N05AB, N05AC, N05AX, N05AE, N05AF, N05AG, N05AH, N05AL |
|  | *Depression* | **2** |  | 296.2, 296.3, 296.82, 298.0, 300.4, 301.12, 309.0, 309.1, 311 | N06A |
|  | *Bipolar disorders* | **2** | 044.296.0, 044.296.1 | 296.0, 296.1, 296.4, 296.5, 296.6, 296.7, 296.80, 296.81, 296.89, 296.9, 298.1 | N05AN |
|  | *Alcohol abuse* | **2** | 014.303, 011.291.1 | 291.1, 291.2, 291.5, 291.8, 291.9, 303, 303.9, 305.0, V11.3 | N07BB01 |
|  | *Drug addiction* | **2** | 014.304 | 292.0, 292.82-292.89, 292.9, 304, 305.2-305.9 | N07BB04 |
|  | *Anxiety* | **2** |  | 300.0 | N05BA, N05BB01, N05CD, N05BC01, N05BC51, N05BX, N05CF, N05CX01, N06BX |
|  | *Other mental disorders* | **2** | 011.294.0, 005.307.1, 005.307.51 | 290.8, 290.9, 291.0, 291.3, 291.4, 292.1, 292.2, 292.81, 293, 294, 299.0, 299.8, 299.9, 300.0-300.2, 300.3, 300.5-300.9, 301.0, 301.10, 301.11, 301.2-301.9, 302, 305.1, 306-308, 309.2-309.4, 310, 312-319 |  |

**Table S2 continued**

| **Diagnostic Categories** | **Disease** | **Vulnerability index** | **Ticket exemption code** | **ICD-9-CM** | **ATC** |
| --- | --- | --- | --- | --- | --- |
| **Diseases of the nervous system and sense organs** | *Parkinson’s disease and Parkinsonism* | **2** | 038.332 | 332 | N04 |
|  | *Multiple sclerosis* | **3** | 046.340 | 340 | L03AB07, L03AB08, L04AA23, L04AA27, L03AX13, L04AA31, L04AA34, L03AB13, L04AX07 |
|  | *Epilepsy and recurrent seizures* | **2** | 017.345 | 345 | N03AF01, N03AB02, N03AA02, N03AA03, N03AA04, N03AE01, N03AD01, N03AG01, N05BA09, N03AG04, N03AX10, N03AG06, N03AF02, N03AX14, N03AX15 |
|  | *Glaucoma* | **1** | 019.365.1, 019.365.3, 019.365.4, 019.365.5, 019.365.6, 019.365.8 | 365 | S01E |
|  | *Other diseases of the nervous system and sense organs* | **3** | 038.333.0, 038.333.1, 038.333.5, 041.341.0 | 320-326, 330-331, 333-337, 340-344 |  |
| **Diseases of the respiratory system** | *Chronic obstructive pulmonary disease, asthma,*  *chronic respiratory disease* | **2** | 007.493, 057 | 490-494, 496 | R03AA, R03AB, R03AC, R03DA, R03DB, R03DA20, R01AC01, R03BC01, R01AC51, S01GX01, S01GX51, R03BA |
|  | *Cystic fibrosis* | **3** | 018.277.0 | 277.0 | R05FB01, R05FA01, A09AA02, R07AX02, R07AX30, R07AX31 |
|  | *Other diseases of the respiratory system* | **3** | 024.518.83 | 472-474, 476, 495, 500-508, 515-519 |  |
| **Diseases of the digestive system** | *Liver cirrhosis and other liver chronic diseases* | **3** | 008.571.2, 008.571.5, 008.571.6, 016.571.4, 016.070.32, 016.070.33, 016.070.54, 016.070.9 | 571, 573, 070 | J05AP08, J05AP09, J05AP51, J05AP53, J05AP54, J05AP55, J05AP56, J05AP57, B05AA01 |
|  | *Inflammatory bowel diseases (Ulcerative colitis and Chron’s disease)* | **2** | 009.555, 009.556 | 555-556 | A07EC01, A07EC02, A07EC03, A07EC04 |
|  | *Chronic and acute pancreatitis* | **2** | 042.577.1 | 577.0-577.1 |  |
|  | *Other diseases of the digestive system* | **2** | 059.579 | 530-538, 557, 572, 567-570, 579 |  |

**Table S2 continued**

| **Diagnostic Categories** | **Disease** | **Vulnerability index** | **Ticket exemption code** | **ICD-9-CM** | **ATC** |
| --- | --- | --- | --- | --- | --- |
| **Diseases of the circulatory system** | *Ischemic heart disease/Angina* | **3** | 0A02.414 | 410-414 | C01DA, C01DX |
|  | *Heart failure* | **3** | 0031.402, 0031.403, 0031.404, 0031.405.0, 021.428 | 398.91, 402-405, 428 |  |
|  | *Arrhythmia* | **2** | 0A02.426, 0A02.427, 0A02.V45.0 | 426, 427, 785.0, V45.0, V53.3 | C01BA, C01BC, C01BD |
|  | *Valvular diseases* | **2** | 0A02.394, 0A02.395, 0A02.396, 0A02.397, 0A02.424, 0A02.V42.2, 0A02.V43.3 | 093.20-093.24, 394-397, 424, 746.3-746.6, V42.2, V43.3 |  |
|  | *Vascular diseases* | **1** | 0C02.440, 0C02.441.2, 0C02.441.4, 0C02.441.7, 0C02.441.9, 0C02.447.1, 0C02.557.1, 0C02.V43.4, 036.443.1 | 440, 441.2, 441.4, 441.7, 441.9, 443.1-443.9, 447.1, 557.1, 557.9, 785.4, V43.4 |  |
|  | *Cerebrovascular diseases* | **3** | 0B02.433, 0B02.434, 0B02.437 | 430-438 |  |
|  | *Hypertension* | **2** | 0A31.401 | 401 | C03AA, C03AB, C03AH, C03AX01, C02CA04, C03BA02, C03BA03, C03BA04, C03BA05, C03BA07, C03BA08, C03BA09, C03BA10, C03BA11, C03DB01, C03DB02, C03EA, C09BA02-C09BA09, C09BB, C09DB, C09DA01- C09DA04, C09DA06-C09DA08, C02AB01, C02AB02, C02AC01, C02AC02, C02AC04, C02AC05, C02DB02- C02DB04, C02DC01, C02DD01, C02DG01, C02KA01, C02KB01, C02KC01, C02KD01, C02KX01, C09XA |
|  | *Coronary and peripheral vascular disease* | **2** |  |  | B01AB, B01AX01, B01AD10, B01AD12, C04AD03, B01AC05 |
|  | *Oral anticoagulant agents* | **2** |  |  | B01AA, B01AE, B01AF |
|  | *Other diseases of the circulatory system* | **2** | 0C02.442, 0C02.444, 0C02.447.0, 0C02.447.6, 0A02.416, 0A02.417, 0A02.429.4 | 390-392, 393, 397.9, 398.90, 398.99, 411.8, 412-417, 420-423, 424.99, 425, 429, 441.0, 441.1, 441.3, 441.5, 441.6, 442, 443.0, 444-446, 447.0, 447.2-447.9, 448 |  |

**Table S2 continued**

| **Diagnostic Categories** | **Disease** | **Vulnerability index** | **Ticket exemption code** | **ICD-9-CM** | **ATC** |
| --- | --- | --- | --- | --- | --- |
| **Diseases of the genitourinary system** | *Chronic kidney disease* | **3** | 023.585 | 585, V451, V56 | V03AE |
|  | *Other kidney disorders* | **2** | 061.581.1, 061.581.2, 061.582.1, 061.582.2, 061.582.4, 061.587, 062.753.13 | 580-584, 586, 587, 588-589, 753,13 |  |
|  | *Other diseases of the genitourinary system* | **1** | 061.590.0, 063.617 | 590-608, 611, 617 |  |
| **Diseases of the skin and subcutaneous tissues** | *Diseases of the skin and subcutaneous tissues, including Not rheumatoid psoriasis* | **1** | 045.696.1, 059 | 690-695, 696.2-696.5, 696.0, 696.1, 696.8 | D05BB01, D05BB02, D05AX |
| **Diseases of the musculoskeletal system and connective tissue** | *Autoimmune disease (rheumatoid arthritis, rheumatoid psoriasis, anchylosing spondylitis, systemic sclerosis, systemic lupus erythematosus)* | **3** | 028.710.0, 054.720.0, 006.714.0, 006.714.1, 006.714.2, 006.714.30, 006.714.32, 006.714.33 | 710.0, 710.1, 714, 720.0 |  |
|  | *Other diseases of the musculoskeletal system and connective tissue* | **2** | 030.710.2, 067.710.9 | 710.2-710.9, 711-713 |  |
| **Other conditions** | *Transplantation* | **3** | 050, 052.V42.0, 052.V42.1, 052.V42.6, 052.V42.7, 052.V42.8, 052.V42.9, 053.V42.5 | V42 | L04AA01-L04AA06, L04AA08-L04AA12, L04AA14- L04AA19, L04AA21, L04AD01, L04AD02, L04AX01 |
|  | *Chronic pain* | **1** |  | 338.2, 338.4 | N02AG01, N02AE01, N02AB03, N02AA05, N02AA55, N02AA03, N02AX06 |
|  | *Corticosteroids* | **2** |  |  | H02 |
|  | *Hormone therapy (oral contraceptives or replacement hormone therapy)* | **1** |  |  | G03AA, G03AB, G02BB01, G03HB01, G03CA |
| **Rare conditions** | *Rare conditions (Rare diseases, physical, mental or intellectual disabilities, and complex multiple diseases)* | **3** | RX, 049, 051 |  |  |

**Table S3 - Effect of Vaccination and Infection on the Risk of New Infection and Hospitalization. Multivariate Conditional Logistic-Regression Analysis, Adjusted by Vulnerability Index**

| **Variables** | **Risk of Infection** | | | **Risk of Hospitalization** | | |
| --- | --- | --- | --- | --- | --- | --- |
|  | **All Period** | **Until**  **30/11/2021** | **After**  **01/12/2021** | **All Period** | **Until**  **30/11/2021** | **After**  **01/12/2021** |
|  | **OR (95%CI)** | **OR (95%CI)** | **OR (95%CI)** | **OR (95%CI)** | **OR (95%CI)** | **OR (95%CI)** |
| **Immunization status** |  |  |  |  |  |  |
| Unvaccinated and not previously infected | 1 (ref) | 1 (ref) | 1 (ref) | 1 (ref) | 1 (ref) | 1 (ref) |
| Vaccinated and not previously infected | 0.64 (0.62-0.66) | 0.43 (0.38-0.49) | 0.67 (0.64-0.69) | 0.10 (0.07-0.14) | 0.12 (0.07-0.22) | 0.08 (0.05-0.13) |
| Unvaccinated and previously infected | 0.35 (0.30-0.40) | 0.11 (0.08-0.17) | 0.55 (0.46-0.65) | 0.10 (0.04-0.28) | 0.10 (0.03-0.32) | 0.07 (0.01-0.76) |
| Vaccinated and previously infected | 0.37 (0.34-0.41) | 0.21 (0.12-0.34) | 0.40 (0.36-0.44) | 0.02 (0.00-0.13) | NE | 0.02 (0.00-0.16) |
| **Vulnerability index** |  |  |  |  |  |  |
| 0-1 | 1 (ref) | 1 (ref) | 1 (ref) | 1 (ref) | 1 (ref) | 1 (ref) |
| 2 | 1.07 (1.04-1.11) | 1.09 (1.01-1.17) | 1.07 (1.03-1.11) | 1.65 (1.36-2.00) | 1.52 (1.23-1.88) | 2.51 (1.54-4.09) |
| 3 | 1.11 (1.07-1.17) | 1.17 (1.07-1.29) | 1.10 (1.04-1.16) | 2.04 (1.65-2.52) | 1.90 (1.50-2.41) | 2.79 (1.69-4.62) |

NE: not estimable
